# Approaches to developing the capacity of health policy analysis institutes: a comparative case study

**DOI:** 10.1186/1478-4505-10-7

**Published:** 2012-03-05

**Authors:** Sara Bennett, Adrijana Corluka, Jane Doherty, Viroj Tangcharoensathien

**Affiliations:** 1 Johns Hopkins School of Public Health, 615 North Wolfe St, Baltimore, USA; 2 Independent researcher and part-time lecturer, School of Public Health, University of the Witwatersrand, 1 Jan Smuts Avenue, Braamfontein, South Africa; 3 International Health Policy Program, Ministry of Public Health, Tiwanond Rd, Nonthaburi 11000, Thailand

## Abstract

**Objectives:**

To review and assess (i) the factors that facilitate the development of sustainable health policy analysis institutes in low and middle income countries and (ii) the nature of external support for capacity development provided to such institutes.

**Methods:**

Comparative case studies of six health policy analysis institutes (3 from Asia and 3 from Africa) were conducted. In each region an NGO institute, an institute linked to government and a university based institute were included. Data collection comprised document review, semi-structured interviews with stakeholders and discussion of preliminary findings with institute staff.

**Findings:**

The findings are organized around four key themes: (i) Financial resources: three of the institutes had received substantial external grants at start-up, however two of these institutes subsequently collapsed. At all but one institute, reliance upon short term, donor funding, created high administrative costs and unpredictability. (ii) Human resources: the retention of skilled human resources was perceived to be key to institute success but was problematic at all but one institute. In particular staff often moved to better paid positions elsewhere once having acquired necessary skills and experience, leaving remaining senior staff with heavy workloads. (iii) Governance and management: board structures and roles varied according to the nature of institute ownership. Boards made important contributions to organizational capacity through promoting continuity, independence and fund raising. Routine management systems were typically perceived to be strong. (iv) Networks: linkages to policy makers helped promote policy influences. External networks with other research organizations, particularly where these were longer term institutional collaborations helped promote capacity.

**Conclusions:**

The development of strong in-country analytical and research capacity to guide health policy development is critical, yet many health policy analysis institutes remain very fragile. A combination of more strategic planning, active recruitment and retention strategies, and longer term, flexible funding, for example through endowments, needs to be promoted. Specific recommendations to funders and institutes are provided.

## Background

In-country capacity to direct health spending, guide implementation, and monitor and evaluate progress is critical if recent global investments in health systems and priority health conditions are to reap their full benefits. Such local analytical capacity is more likely to lead to local ownership of findings and the uptake of research evidence in policy decisions, through closer connections between researchers and policy makers [1]. It may also promote more timely responses to emergent challenges in dynamic health systems [2]. Furthermore, reliance upon technical support and analysis from high income, donor countries is typically costly and may be ineffective [3]. Consequently, investing in capacity development for local research institutes and think tanks is emphasized in the policies of many international agencies [4] and declarations [5].

Accordingly a major international program of support for policy analysis institutes, the "Think Tank Initiative", was launched in 2008 to support the development of think tanks in low and middle income countries [6,7]. Within the health sector the growing appreciation of the potentially important benefits associated with health policy analysis institutes (HPAIs), has led several donors, including the World Bank, the World Health Organization (WHO) and the UK Department for International Development (DFID) to establish and support HPAIs in countries as diverse as Uganda, Kyrgyzstan, and Bangladesh. Overall there appears to have been a recent proliferation of analytical organizations of this nature including observatories, think tanks and knowledge translation platforms. Initial work conducted for this study identified a total of 78 HPAIs in low and middle income countries, over 80% of which had been established since 1990.

Despite these developments, and despite the fact that many reports have highlighted significant differences in research capacity between high income countries, and low and middle income countries [8-10], relatively few studies have sought to understand the factors affecting the development of capacity for analysis and research, nor what constitutes good practice in this field. This is particularly true for health policy and systems research [11].

The literature on the impact of investments on the capacity of policy analysis institutes in low and middle income countries is equally limited and that which is available is not specific to health. One study assessing the impact of the Open Society Institute's International Policy Fellowship program found that the program had positive effects on individual beneficiaries but broader impacts on strengthening policy formulation were mixed [12]. More encouragingly, an assessment of the Policy Research Organization Project in Bosnia and Herzegovina concluded that the investment had had beneficial effects, replacing the dominance of the international community in policy debates with greater reliance on local policy research organizations [13].

The available evidence does suggest however that attracting and retaining high quality staff is a key challenge for many policy analysis institutes. Researchers have debated what constitutes a critical mass of professional staff [14-16]. Regular rotation of staff from policy analysis institutes into government and back, has been observed to help maintain a strong policy orientation in the institutes [16,17]. Other authors have explored the nature of funding for policy analysis institutes and in particular the problems associated with short term contracts, including the constant treadmill of proposal writing and contract negotiations, and also the loss of ability to respond to fleeting windows of political opportunity in the policy agenda due to lack of flexible funding [18].

Our research was initiated to explore these issues further and aimed to (i) describe and analyse the different dimensions of organizational capacity in HPAIs in low and middle income countries and (ii) draw lessons about the nature of appropriate external support for capacity development of such institutes. A companion paper reports on the effectiveness of the HPAIs in influencing policy development [19]. For the purposes of this study, an HPAI was understood to have the overall purpose of supporting health policy development and implementation through analysis and research, with health policy makers as its primary clients. In our view such institutes might take different organizational forms, but typically possess some degree of autonomy and are not profit oriented. HPAIs may perform a range of different functions including conducting policy-relevant research and analysis, providing policy advice and technical assistance in policy formulation and evaluation, conducting policy dialogues and training policy-makers.

The focus of this paper is on organizational capacity. The UK Department for International Development (DFID) defines organizational capacity as "the capacity of research departments in universities, think tanks and so on to fund, manage and maintain themselves" [20]. There are many different frameworks that help to conceptualize the different aspects of organizational capacity [9,21], often these share some common features. We identify three broad dimensions of organizational capacity:

• Resources -particularly financial resources and staff, and sustainability

• Governance and management - comprising both external accountability mechanisms (such as boards) and internal management systems

• Networks - relationships with other organizations that assist in achievement of organizational goals.

These dimensions of organizational capacity are used to structure the results section of the paper.

## Methods

A comparative case study approach was used as it provides a structured approach to studying complex causal relationships through the in-depth study of a limited number of cases. A complete listing of HPAIs in low and middle income countries was developed drawing upon existing sources (such as databases held by the Global Development Network and the Alliance for Health Policy and Systems Research) as well as a web-based search. Basic information on all of the HPAIs in the database was compiled. HPAIs for this study were purposively selected from the compiled list. According to the brief provided by the research funder, the Rockefeller Foundation, we focussed on Asia and Sub-Saharan Africa although future research should undoubtedly examine the invaluable experience in other parts of the world, especially Latin America. Within our chosen region we sought to maximize the variability in cases [22] while also identifying institutes willing to participate in the study. Specifically we sought to study one non-governmental organization (NGO), one university and one government owned policy analysis institute in both Asia and Africa and also to select cases that had received differing levels of external support. All of the case study institutes had an explicit focus on health sector issues. The list of institutes studied is shown in Table 1 and includes: Health Strategy and Policy Institute (HSPI), Vietnam; Health Economics Unit (HEU), South Africa; Institute for Health Systems (IHS), India; Health Economics Institute (HEI), Bangladesh; Health Policy Analysis Unit (HPAU), Uganda; and the Center for Health and Social Services (CHeSS), Ghana.

**Table 1:** Profile of case study institutes and initial support received

Institute & Country	Legal Status	Establishment	External support to capacity development	Current Situation
Health Strategy and Policy Institute (HSPI), Vietnam	Public entity under jurisdiction of MOH	1998 in its current form - evolved from previous government organizations.	Initial support primarily from government	Regarded as an effective player in informing policy debates nationally

Health Economics Unit (HEU), South Africa	Formally established institute within the School of Public Health and Family Medicine, University of Cape Town	1990 - established by previous head of the Unit with support from the University of Cape Town.	Limited outside support received initially	Well established and well respected both nationally and internationally

Institute for Health Systems (IHS), India	NGO, registered as a society	1990 - established by senior civil servant who acted as head of the Institute for several years.	Limited outside support received initially	Has had many changes in fortune, currently re-establishing itself after financial difficulties

Health Economics Institute (HEI), Bangladesh	Formally established institute within Department of Economics, University of Dhaka	1998 - established by government with support from UK DFID	UK DFID grant for 5 years, with a value of US$194,000 per annum. Included support for organizational development, staff training and capacity development, and financial support	Receives minimal funding and health policy analysis functions have dramatically declined. The institute now plans to broaden its mission and mandate beyond the health sector.

Health Policy Analysis Unit (HPAU), Uganda	Integral to MOH	1999 - established by government with support from the World Bank	World Bank support of US$245,000 per annum for five years, designated largely for policy analysis work	Receives minimal funding, and its position within the MOH hierarchy has declined

Centre for Health and Social Services (CHeSS), Ghana	Registered NGO	2008- established by former senior civil servant	Grant from Rockefeller Foundation, US$394,000 per annum provides support for organizational development as well as technical work	Still in early phases of development

Case studies were conducted in 2009 by researchers from the country or region concerned, who were familiar with the institutes but were not staff members at them. A common protocol and semi-structured questionnaire were developed to guide data collection in each country. Researchers conducted document reviews of published and unpublished material relating to the institute, and also extracted data from financial records. Guidelines on the selection of key informants was provided in the common protocol: informants were sought from a range of different stakeholders in each country including founders of the institute, staff members, funders, members of the institute board and clients of the institute including policy makers and civil society. An initial list of respondents was based upon document review and a snowball approach used to identify additional respondents. See Table 2 for a final categorization of interviewees by country. Interviews were conducted face-to-face except for a few interviewees who had moved to distant locations and were interviewed by phone or who responded by email. In each country a draft report was discussed with staff members of the institute prior to finalization.

**Table 2:** Respondents by Country

Category of Respondent	HEI Bangladesh	CHeSS Ghana	IHS India	HEU South Africa	HPAU Uganda	HSPI Vietnam
Staff of Institute	6	2	6	6	3	11

MOH/government	3	4	7	3	4	4

Other stakeholder*	4	1	5	3	6	2

Total	13	7	18	12	13	17

*Includes board members, funders and other clients of the institute: composition varies according to context

In most cases interview data were transcribed, in some instances interviews were not recorded but detailed notes were made of the interviews. During analysis, researchers sought in the first instance to provide factual descriptions of the evolution of the institute over time, including mapping financial and staffing trends, and conducting an institutional analysis of organizational structure, governance and leadership. Further analyses were more explanatory and sought to explore respondents' perspectives on sustainability and capacity development in particular. Analyses combined the document review, interview data and feedback from respondent validation processes. Interview data were not coded but the researchers sought to extract relevant data from interviews based on the key themes in the protocol (including for example, the HPAI's contextual environment, organizational structure and governance mechanisms, human resources, financial resources, research portfolios, and experience in policy making, among others). Firstly, reports on each institute were developed by the respective case study authors. These reports, together with supplementary primary data, were used to develop the final synthesis of findings presented in this paper. Ethical approval was obtained from the WHO ethics committee. At the country level, the local research team sought local advice about the need for ethical review. Full ethical review was received in three countries (Ghana, India and Uganda); in the remaining three countries local stakeholders advised that ethical review was not required. In all countries the research was approved by the institute director.

## Results

### Financial Resources

Of the six case study institutes, three (the HEI, Bangladesh; HPAU, Uganda and CHeSS, Ghana) received substantial external support (in the range of US$200,000-400,000 per annum) at start up. World Bank support to the HPAU, Uganda was primarily for technical work and had only very limited support for capacity development. In contrast the HEI, Bangladesh received funding from DFID that included support for staff training, the purchase of equipment, and infrastructure development, including the establishment of a resource center. One PhD and seven Masters students were trained through this facility. CHeSS, Ghana also received significant financial support from the Rockefeller Foundation during its start-up phase for capacity development, however it is too soon to determine the effects of this upon the organization.

In Uganda and Bangladesh, the cessation of donor support created major difficulties for the respective institutions. In Uganda the HPAU had only limited success in attracting additional funding after World Bank funding finished, and increases in government funding were insufficient to keep the institute functional. Similarly in Bangladesh, HEI was still highly dependent on DFID funding when the funding ended and was unable to sustain itself from other sources.

*"Where HEI failed - or perhaps where the [consulting firm] support did not have sufficient time to support capacity building, was in building capacity within the university to mobilise research and other funds from a variety of different sources. Had the university developed a consulting capacity, there would have been scope for the sort of income generation that is needed to keep this type of unit functioning at a high level, but the level of dependency on the DFID funding was too high, so when the funds were withdrawn..... then there was insufficient capacity to mobilise funds from other sources." *(External stakeholder, Bangladesh)

DFID funding to HEI ceased earlier than had originally been anticipated due to a complex set of reasons, including issues of donor aid management, political transitions, institutional rivalry and financial transparency. However the root cause was a change in government, resulting in a regime that was no longer strongly supportive of the health reforms or the role of evidence in driving these reforms, and ultimately was not supportive of the HEI. Unfortunately the limited life of DFID support to HEI and the hostile policy environment that ensued, prevented earlier investments from coming to full fruition.

As noted above, funding at the HEI, Bangladesh and the HPAU, Uganda diminished to levels so low that these institutes were no longer fully functional. While funding for other institutes was better, it was typically modest, and these institutes were still perceived by both internal and external stakeholders to be financially vulnerable. For example, forty-five percent of HEU, South Africa's operating budget was a grant mainly for regional capacity-building which came to an end in 2010. Only HSPI, Vietnam had an operating budget greater than US$1 million per annum (Table 3).

**Table 3:** Funding of Case Study Institutes

Source of Funding	HEI Bangladesh (2008) (%)	CHeSS Ghana (2009) (%)	IHS India (Avg 2003 - 2009) (%)	HEU South Africa (avg. 2004- 2009) (%)	HPAU Uganda 2008/09 (%)	HSPI Vietnam (Avg 2004-2009) (%)
Multilateral agencies (EU, WHO)	0	16	22	12	0	18

Bilateral agencies (SIDA, DFID)	0	15	18	67	0	11

Private foundations	0	58	20	0	0	13

Government (national/provincial)	0		36	6	100	58

NGO (local and international)	0	0	0	7	0	0

Academic/research organisations	100	11	5	8	0	0

Approximate Annual Budget (in US$) (where two figures are given this indicates a range of annual funding)	66,500	680,000	92,871 -178,500 during past three years	790,000	26,000	1,300,000 (2007)

HSPI, Vietnam, equivalent to a department of the MOH, was the most financially secure: it receives substantial funding from the Ministry of Health (about 60% of total revenues) which covers all basic salary costs, with salary top-ups provided from external projects. The other functional institutions did not receive core financial support from government. In South Africa, the HEU does a substantial amount of advisory work for government, but very little is remunerated as the bureaucratic hurdles in establishing and operating contracts with government were viewed as too cumbersome to typically be worthwhile.

While it might appear that HPAIs hosted by other institutions (such as universities) might gain some financial sustainability from this relationship, in practice the financial responsibilities of the host institution were frequently limited. The HEU, South Africa for example, only received university funding for the Director's position and was otherwise reliant on soft-funding. Funding received by most HPAIs was thus largely project-specific and linked to particular deliverables. Institutes had found it challenging to attract and retain flexible, core, funding. For example, the IHS, India had attempted a few years ago to launch a new Masters program with a view to establishing a steady and reliable revenue stream. The institute had made substantial start-up investments in this program but due to issues regarding agreements on accreditation, the course was never properly launched and this venture undermined the financial viability of the institute for several years.

Proliferation of multiple small scale projects was problematic. For example, HSPI Vietnam's research portfolio from 2005 to 2009 comprised a total 64 projects, with 70% being short-term projects of less than one year. This led to a high administrative burden. Similarly, observers of the HEU, South Africa thought that substantive effort was expended by the Unit to secure relatively limited amounts of funding.

*"They survive but they spend a lot of time chasing money......it's just silly that a Unit as successful as this has to spend so much time looking for a bit of money here and a bit of money there" *(External Observer, South Africa)

Staff at the HEU, South Africa noted that initially they had been almost entirely reliant on small, short-term project funding, but had managed over time to secure a number of larger, longer term programs that provided greater financial security. While the HEU, South Africa is strongly committed to internal capacity development it has received only limited funds dedicated to this purpose and these only materialized relatively late in its development. Furthermore, it is frequently viewed by external partners as a mechanism through which to build regional capacity rather than needing such support itself.

None of the institutes had a clear financial or fund-raising strategy, and none had professionalized the fund raising role. Researchers and policy analysts undertook fund-raising as an "add on" to their core roles. While some institutes were relatively sophisticated in terms of understanding and responding to the funding environment, fund-raising at many institutes remained passive and lacked strategic direction.

#### Human resources

With the exception of CHeSS, Ghana (which is still relatively young and facing an unpredictable workload), all of the institutes relied primarily on in-house research staff, though external consultants were sometimes used to fill particular gaps.

Table 4 shows the number of staff by qualification. The question of how to attract and retain well qualified staff was perceived to be a key challenge particularly for the HEU, South Africa and the IHS, India. The IHS has struggled to hire and retain senior and experienced health researchers: only four of the current staff have substantive (at least 3-4 years) research experience. Interviews with current and past staff members found that as researchers gained in experience and recognition, the financial position of the IHS could not provide them with financial security and so they left for better paying jobs. Equally importantly, the small pool of senior staff remaining in the organization carried a heavy burden including fund-raising, mentoring new researchers and overseeing an increasing number of small grants while having insufficient opportunities to specialize and develop their own skills. Burn-out of good researchers was a major problem faced by the Institute. As one former staff member said:

**Table 4:** Staffing at case study institutes, 2009

Professional staff by highest degree	HEI Bangladesh	CHeSS Ghana	HIS India	HEU South Africa	HPAU Uganda	HSPI Vietnam
PhD	5	1	0	4	0	7

Masters	6	1	12	5	1	14

Bachelors	0	3	4	1	0	9

Administrative & other staff	4	2	10	4	0	12

**TOTAL**	15	7	26	14	1	42

*"When I was there, I worked long hours and enjoyed it; but then I found it difficult to carry on due to financial and family needs"*. (Former staff member, IHS, India)

Recruitment of senior staff was also identified as a critical issue at the HEU, South Africa. The institute had not been able to recruit an additional person with a PhD for seven years prior to the study. Respondents attributed this to the global shortage of health economists (particularly senior people with experience in low and middle income countries) combined with the University salary structure. The University has a relatively compressed salary structure meaning that junior researchers are well paid compared to government, but senior people earn salaries that, until very recently, were 40% lower than market value. As a consequence, the HEU has found itself mentoring and training a large number of more junior people, who having gained experience, then leave to better paid positions in government. Like the IHS, the difficulties in hiring more senior level staff mean that existing senior staff bear a very heavy workload, and there is a real danger of burnout.

Problems of staff retention at the HSPI, Vietnam appeared less significant than elsewhere. All the staff trained under the long-standing Vietnam-Sweden Research Cooperation Programme had returned to serve in Vietnam. HSPI staff are not highly paid but non-financial incentives are important. Low staff turnover was attributed by respondents to high morale and commitment: staff said they are well recognized by high level officers, proud of their work and their contribution to society, have a good working environment, and are adequately paid. Respondents at the HEU, South Africa also noted that the very positive work culture at the institute made it an appealing and supportive place to work.

#### Governance and management

Governance structures at the institutes differed according to the nature of institute ownership. The two NGOs, CheSS, Ghana and IHS, India both had their own Boards. The HEI, Bangladesh was self-governing and reported to the syndicate of the university through its own Board of Governors. However the other university-based institute, the HEU, South Africa had no independent advisory or governance structure but was accountable through the usual university channels. Respondents at the HEU perceived the governance arrangements to be satisfactory, although stable leadership of the Department in which the HEU is located and good personal relations amongst Departmental staff had clearly played a part in making this arrangement effective.

For the two institutes based in Ministries of Health (HSPI, Vietnam and HPAU, Uganda) issues related to management and governance appeared critical: neither institute had a separate governing body and both reported directly through ministry channels. Originally, the HPAU in Uganda was created as an independent unit reporting directly to the Permanent Secretary, well above the heads of departments. This reporting structure was intended to give the unit autonomy and power to influence policies coming from technical departments. However many officials senior to the head of HPAU were effectively bypassed by this arrangement, and given the bureaucratic and hierarchical nature of government this proved problematic. Initially staff managed to make the arrangement work through employing informal channels of communication, but as the resources available for HPAU support declined, the unit's authority was undermined and its level within the bureaucratic hierarchy downgraded through organizational reforms.

HSPI, Vietnam does not have a Board but it has a Scientific Committee (responsible for maintaining quality standards) and an Advisory Committee responsible for strategic direction. Some respondents suggested however that both these committees were dominated by government officers, and consequently the ability of HSPI to provide truly independent advice was circumscribed.

In contexts where strong boards were in place they were perceived to play a very positive role. At IHS India, while the Board only meets once a year, Board members often interact with individual staff members on a one-to-one basis in the interim, and respondents stated that the Board was critically important both in terms of setting long term strategy, and with respect to fund raising. IHS has a complex board structure with both charter members of the board and regular members. Of the original founding charter members in 1990, seven continue in their roles thus providing a substantial degree of stability for an entity that has suffered from relatively high staff turnover.

In none of the institutes were routine management systems perceived by respondents to be problematic, but host institutions' regulation of reimbursement was thought to be problematic in cases where the institute was dependent upon the management policies of a parent organization. In Vietnam, Bangladesh, Uganda and South Africa the prevailing salary levels were thought to be too low to attract and retain qualified staff. Vietnam addressed this by using grants with external agencies to "top up" salaries. The HEU, South Africa was also exploring a special university provision allowing certain professions to be paid "above-rate-for-job" and recently succeeded in negotiating this.

#### Organizational networks

Within their country contexts, the more successful institutes benefitted from strong links to policy makers which affected not only their ability to influence policy, but also supported their research capacity. As discussed in the companion article [19], frequently these links were derived from the individual reputations of institutional leaders. At the HSPI, Vietnam there were concerns about how best to institutionalize these networks. HEU, South Africa also works closely with another South African policy analysis institute, the Center for Health Policy, which has enabled both institutes to participate in, and even lead, much larger, multi-partner projects than would have been possible on their own.

Three of the institutes - HSPI, Vietnam; HEU, South Africa; and IHS, India - had benefitted from longer term institutional collaborations with organizations outside their own country. These linkages had been particularly beneficial for capacity development. HSPI was a key beneficiary of the Vietnam-Sweden Research Cooperation Programme that has been active since the 1990s. In addition to training, this program helped build institutional links with the Karolinska Institute in Stockholm. Staff at the HEU in South Africa also noted the importance of linkages to universities outside of the country. Strong and long term links had been cultivated with the London School of Hygiene and Tropical Medicine (LSHTM) and the Karolinska Institute. As the relationship with LSHTM has evolved into a broader consortium, HEU's role has also evolved into one which both benefits from and contributes to capacity development efforts to countries in the region. IHS, India has multiple links and partnerships, although the majority of these are with in-country institutions. At the HEI, Bangladesh, university politics proved difficult to navigate, resulting in few networks and few links to other universities being established.

While HSPI Vietnam has strong links to the Karolinska Institute and strong professional networks with researchers at provincial and district levels within Vietnam, it seemed that its international research networks are relatively narrow and ad hoc. Respondents recognized the importance of developing such networks:-

*"There should be an official way of collaboration, maintaining and sustaining such collaborations, wider partners and more official arrangement. Now HSPI invites them on a project basis, [there is] no continuity and [it is] not sustainable. HSPI need more hands, and spend time for thinking and policy interface." *(government official, Vietnam)

## Discussion

This study approached the question of how to facilitate the development of sustainable independent policy analysis institutes in the health sector, from a fairly broad perspective, taking account of financial and human resources, governance and management issues, and networks.

While our initial thinking was that HPAIs may face challenges quite different from standard research organizations or universities, given both their stronger focus on policy engagement, and their more independent organizational forms, in fact many of the challenges that we identified resonate with the broader literature on research capacity in LMICs. As other papers have done before us, we identified the problems created by small, short term grants and the disproportionate effort associated with securing such grants[23]; the challenges to securing appropriate levels of staff remuneration, particularly for more senior staff [24]; the need to create effective south-south and north-south partnerships and networks [23-25], and the required long term nature of commitment required for effective and sustainable capacity development [25,26].

The ability to attract and retain senior staff was found to be critical to the success of health policy analysis institutes. Such senior staff lend credibility to communications with policy makers, bring significant personal networks to the institute, guide the program of technical work, mentor junior staff and are critical to fund raising efforts. Yet it is particularly among this cadre that issues of burn-out and relatively low salaries appeared most problematic. Capacity development initiatives need to recognize the critical role of senior staff, and explore ways to attract and retain them (see How to Promote Capacity and Sustainability within HPAIs). Three of the case study institutes had been established by individuals who were well known and committed in their field. In such circumstances succession planning and the development of leadership skills among other staff are key to institute sustainability.

Based upon our findings we propose a set of strategies that HPAIs should pursue so as to promote their own development and sustainability (see How to Promote Capacity and Sustainability within HPAIs).

The findings from this study also resonate more widely with the broader development literature on developing sustainable institutions [27,28]. These writings draw attention to the need to think about sustainability not only in terms of internal capacities, but also in terms of the receptivity of the external environment to the institute. In particular factors such as the stability of the external environment, the openness to change and above all the demand for the services of the institute are identified as key to institutional sustainability. From the case studies conducted here, particularly those in Bangladesh and Uganda, it appears not uncommon that unanticipated shifts can rapidly turn an external environment from hospitable to hostile. Prior to investing in an HPAI, funders need a realistic assessment of the true demand for services of the HPAI from country level stakeholders, and the environmental threats that it faces.

While it is unlikely that HPAIs can manipulate their external environment for the better, it is critical that they (and their supporters) understand environmental constraints and approach them strategically. In particular, analysts of institutional sustainability have argued for the importance of organizational learning so that the institute can proactively position itself within a rapidly changing environment, rather than passively reacting to new developments [27,29]. In light of this, institute Boards may have a critical role to play in promoting learning and strategic planning, and bringing intelligence from the outside world. In addition to understanding technical issues, board members need to bring strategic thinking skills as well as extensive personal networks that can help garner strategic information. Tools and approaches that facilitate strategic thinking about organizational development, such as those developed by IDRC as part of the "Think Tank Initiative", may be helpful in this respect [7].

### How to Promote Capacity and Sustainability within HPAIs

• Develop financial plans and a clear fund-raising strategy, pursue diversification of funding sources and longer term program grants, but seek to avoid multiple low value short term contracts which typically carry high administrative costs.

• Invest in developing flexible and predictable funding that enables the institute to build its own program of work, pursue institutional development and respond to unfunded government requests - endowment funding that allows the institute to run on the interest stream from a capital investment may be particularly promising.

• Seek core funding from government annual budget/grants, but avoid excessive reliance on government funding as this may undermine institutional autonomy.

• Develop and make active use of strong Board structures - such structures can help protect the independence of the institute, promote continuity (in the face of staff turnover) and help ensure strategic thinking and learning.

• Ensure that the institute engages in structured systematic thinking, for example through regular assessment of organizational Strengths, Weaknesses, Opportunities and Threats (SWOT analysis) and strategic planning processes - these may be critical to managing unpredictable and dynamic environments.

• Seek innovative ways to attract and retain senior staff including options such as salary top-ups, sabbatical leave and exchange programs, and professional development opportunities.

• Engage early in succession planning and the development of leadership skills across institute staff

• Proactively and strategically expand the networks of the HPAI both domestically and internationally.

## Conclusions

The development of strong research and analysis institutions that can help guide and strengthen health systems in low and middle income countries has been relatively neglected by research funders, international agencies and national governments, although more recently there appears to be growing interest in this field. Health system strengthening requires investment in technically sound, scientifically credible institutions with some measure of independence. Health policy analysis institutes are one type of organization that can help promote health systems strengthening through research, analysis and policy advice. Currently many health policy analysis institutes in low and middle income countries remain rather fragile structures, with limited financial security. Greater strategic planning by the institutes themselves, combined with more savvy external support that combines longer term, flexible funding with clear incentives to diversify institutional resources is needed.

## Competing interests

The authors declare that they have no competing interests.

## Authors' contributions

SB conceived of the study. SB and AC developed the study protocol and instruments. JD and VT led data collection and analysis efforts for the case studies in South Africa and Vietnam. All authors participated in the drafting of the manuscript and all authors read and reviewed the final version of the manuscript.

## References

[B1] Innvaer S Health policy-makers perception of their use of evidence: a systematic revie J Health Serv Res Pol 2002 7 4 239 244 10.1258/135581902320432778 12425783

[B2] Paina L Peters DH Understanding pathways for scaling up health services through the lens of complex adaptive systems Health Policy Plan 2011 first published online August 5, 2011 doi:10.1093/heapol/czr054. 10.1093/heapol/czr054 21821667

[B3] Easterly W Are aid agencies improving Economic Policy 2007 22 52 633 678 10.1111/j.1468-0327.2007.00187.x

[B4] DFID DFID Research Strategy: 2008-2013 2008 London: DFID

[B5] World Health Organization Bamako Call to Action on Research for Health in Global Ministerial Forum on Research for Health: Bamako 2008 Mali: WHO

[B6] McGann JG Best practices for funding and evaluating think tanks and policy research Prepared for the William and Flora Hewlett Foundation 2006 Ambler: McGann Associates

[B7] IDRC The Think Tank Initiative in Review 2010 Ottawa: International Development Research Center

[B8] World Health Organization World Report on Knowledge for Better Health 2004 Geneva: World Health Organization

[B9] Green A Bennett S Sound choices: enhancing capacity for evidence-informed health policy 2007 Geneva: WHO

[B10] Nuyens Y No Development without Research: A challenge for research capacity strengthening 2005 Geneva: Global Forum for Health Research

[B11] Bennett S Building the field of health policy and systems research: an agenda for action PLoS Med 2011 8 8 p. e1001081 doi:10.1371/journal.pmed.1001081 10.1371/journal.pmed.1001081 PMC3168867 21918641

[B12] Pop D Policy Research Fellowships as Knowledge Creation Endeavors: Case study of the Open Society Institute's International Policy Fellowship Program 2005 Budapest: Centre for Policy Studies, Central European University

[B13] Struyk RJ Kohagen K Miller C Were Bosnian policy research organizations more effective in 2006 than in 2003? Did technical assistance play a role Policy Adm Dev 2007 27 5 426 438

[B14] James S Stone D Influencing government policy making Banking on Knowledge: The Genesis of the Global Development Network 2000 Warwick: Warwick Universityl

[B15] Struyk RJ Managing Think Tanks: Practical Gudiance for Maturing Organizations 2002 Budapest: Open Society Institute

[B16] Dror Y Required break throughs in think tank Policy Sci 1984 16 3 199 225 10.1007/BF00138510

[B17] Osman M Nolla NE The politics of independence: can government think tanks act independently? The International Conference on the role of think tanks in developing countries: challenges and solutions 2009 Cairo

[B18] Ali SM Sustainable Development Policy Institute: Pakistan's case study 2005 Islamabad: Sustainable Development Policy Institute 1621887481

[B19] Bennett S Influencing policy change: the experience of health think tanks in low and middle income countries Health Policy and Planning, forthcoming 10.1093/heapol/czr035 PMC3328921 21558320

[B20] DFID Research Programme Consortia: Guidance note on capacity development 2009 London: UK government D.f.I. Development, Editor

[B21] Lusthaus C Adrien M-H Perstinger M Capacity Development: Definitions, Issues and Implications for Planning, Monitoring and Evaluation Universalia Occassional Paper 1999 Montreal: Universalia

[B22] Gerring J Case study research: Principles and practice 2007 Cambridge: Cambridge University Press

[B23] Gonzalez-Block MA Mills A Assessing capacity for health policy and systems research in low and middle income countries Health Res Policy Syst 2003 1 1 1 10.1186/1478-4505-1-1 12646072 PMC151554

[B24] Nchinda TC Research capacity strengthening in the South Soc Sci Med 2002 54 11 1699 711 10.1016/S0277-9536(01)00338-0 12113452

[B25] Mayhew SH Doherty J Pitayarangsarit S Developing health systems research capacities through north-south partnership: an evaluation of collaboration with South Africa and Thailand Health Res Policy Syst 2008 6 8 10.1186/1478-4505-6-8 18673541 PMC2559830

[B26] Lansang MA Dennis R Building capacity in health research in the developing world Bull World Health Organ 2004 82 10 764 70 15643798 PMC2623028

[B27] Brinkerhoff D Goldsmith A Promoting the sustainability of development institutions: A framework for strategy World Development 1992 20 3 360 383

[B28] Gustafson DJ Developing sustainable institutions: lessons from a cross-case analsyis of 24 agricultural extension programme Pub Admin Dev 1994 14 2 121 134 10.1002/pad.4230140202

[B29] Johnson H Wilson G Institutional sustainability as learning Dev Prac 1999 9 1/2 43 55

